# The impact of suicide beliefs on support for suicide prevention and physician-assisted suicide

**DOI:** 10.1186/s12888-025-07112-8

**Published:** 2025-07-09

**Authors:** Nina Ming-Hsin Hsu, Ying-Yeh Chen, Shu-Sen Chang, Ying-Chen Chi, Kevin Chien-Chang Wu

**Affiliations:** 1 Taipei City Psychiatric Centre, Taipei City Hospital, Taipei City, Taiwan; 2 Institute of Public Health, School of Medicine, National Yang Ming Chiao Tung University, Taipei City, Taiwan; 3 Institute of Health Behaviors and Community Sciences and Global Health Program, College of Public Health, National Taiwan University, Taipei City, Taiwan; 4 Psychiatric Research Center, Wan Fang Hospital, Taipei Medical University, Taipei City, Taiwan; 5 Population Health Research Center, National Taiwan University, Taipei City, Taiwan; 6 Department of Healthcare Information and Management, Ming Chuan University, Taoyuan City, Taiwan; 7 Graduate Institute of Medical Education and Bioethics, National Taiwan University College of Medicine, Taipei City, Taiwan; 8 Department of Psychiatry, National Taiwan University Hospital, No.1 Changde Street, Zhongzheng Dist, Taipei City, 10048 Taiwan

**Keywords:** Suicide, Human rights, Rationality, Suicide prevention, Physician-assisted suicide

## Abstract

**Background:**

In 2019, Taiwan implemented two laws with contrasting implications for suicide-related policies: the Suicide Prevention Act, which mandates government-led interventions to prevent suicide, and the Patient Right to Autonomy Act, which affirms patient rights in end-of-life care, including the refusal of nutrition and fluid that hastens death, but does not explicitly legalize physician-assisted suicide. Public support for legal policies related to these laws may depend on beliefs about suicide. This study examined whether two specific beliefs: (1) that people have the right to die by suicide and (2) that suicide can be a rational act, are associated with support for suicide prevention policies and legal penalties for physician-assisted suicide.

**Methods:**

A nationally representative telephone survey (*N* = 1,087) was conducted in Taiwan in 2020. Two belief items served as key predictors: (1) agreement with the statement “people have the right to suicide” (right-to-suicide belief), and (2) disagreement with the statement “suicide is irrational” (suicide-rationality belief). Logistic regression analyses examined associations between these beliefs and attitudes toward three suicide prevention measures and one policy regarding the legal penalties for physician-assisted suicide. Interaction effects between beliefs were also analyzed.

**Results:**

Support for suicide prevention measures ranged from 85 to 95%, while 29.4% supported legal penalties for physician-assisted suicide in terminally ill patients. The right-to-suicide belief was held by 55.3% of participants and was not significantly related to suicide-prevention attitudes, but was associated with support for penalizing physician-assisted suicide. The suicide-rationality belief, held by 26.3%, was associated with reduced support for suicide prevention policies and greater opposition to penalizing physician-assisted suicide.

**Conclusions:**

While the belief in a right to suicide was common, it did not consistently predict support or opposition to suicide-related laws, suggesting that it may be a broad or ambiguous stance. In contrast, the belief that suicide is rational was linked to clearer, more consistent positions across related policies. Public education and engagement efforts should consider how framing suicide as rational may reduce support for prevention. Approach that incorporates medical, psychological, social, and cultural perspectives may help clarify the boundaries between prevention efforts and respect for patient autonomy.

**Supplementary Information:**

The online version contains supplementary material available at 10.1186/s12888-025-07112-8.

## Introduction

Suicide is a critical global public health issue. According to the World Health Organization [1], approximately 703,000 people die by suicide annually, accounting for 1.3% of all deaths worldwide. The United Nations Sustainable Development Goals (SDGs) aim to decrease suicide rates by 33% before 2030 and advocate for universal prevention strategies such as restricting means to suicide, encouraging responsible media reporting, and increasing mental health literacy and access to mental health care [1]. These prevention strategies often require implementation through national legislation, making legal frameworks an important aspect of suicide prevention.

### Taiwan’s contrasting legal approaches: prevention versus autonomy

In Taiwan, the age-standardized suicide rate was 12.9 per 100,000 population in 2019, significantly higher than the global average of 9.0 per 100,000 population [1, 2]. These statistics underscore the urgency of effective suicide prevention measures in Taiwan. In recent years, Taiwan has implemented two seemingly contradictory pieces of legislation, i.e., the Suicide Prevention Act (2019) and the Patient Right to Autonomy Act (passed in 2016 and enacted in 2019). The Suicide Prevention Act (2019) mandates interventions relating to suicide, including surveillance and research on suicide, restriction of lethal means, penalizing certain inappropriate media reporting of suicide, and protocols for reporting and follow-up visits for individuals who attempted suicide [3]. In East Asian cultures, suicide has historically carried less stigma than mental illness. In some cases, morally justified suicide was even officially praised by ancient regimes. Unlike their Western Christian counterparts, these societies did not criminalize suicide [4]. However, with the adoption of modernized criminal codes, assisted suicide became a punishable offense—Taiwan being no exception. In Taiwan, media coverage of suicide has often been sensationalized with detailed description of suicide methods, a practice that research has shown to undermine suicide prevention efforts [5, 6]. Consequently, the Suicide Prevention Act emphasizes media regulations as a way for suicide prevention.

By contrast, the Patient Right to Autonomy Act emphasizes patient autonomy in medical decision-making, enabling patients with terminal illnesses, in a persistent vegetative state, or in an irreversible coma to withdraw or withhold life-sustaining treatments, artificial nutrition, and hydration [7]. Historically, discourse surrounding death has been culturally taboo in Taiwan, contributing to the predominance of paternalistic familism in end-of-life healthcare decision-making. Within this framework, family members, rather than patients themselves, have traditionally assumed the authority to make critical medical decisions regarding terminal care. Demonstrations of filial piety often involved concealing terminal diagnoses from elderly parents and requesting that physicians pursue all possible life-prolonging measures [8]. However, as Taiwanese society has undergone increasing Westernization, a paradigm shift toward individual autonomy in medical decision-making has gained traction. Advocacy for patients’ rights—such as the right to refuse cardiopulmonary resuscitation and to choose hospice care—led to the enactment of the Hospice and Palliative Care Act in 2000 [8, 9]. This milestone was further strengthened by the passage of the Patient Right to Autonomy Act.

In a strict sense, the refusal of artificial nutrition and hydration has a “quasi-suicidal” effect, even in patients with terminal illnesses, as it will hasten death beyond the disease’s natural course. When healthcare professionals assist in implementing this decision, such assistance may be construed as assisted suicide, which remains a criminal offense in Taiwan. This contradiction between individual rights to death and societal responsibility for suicide prevention demonstrates complex ethical considerations around suicide prevention.

### Ethical frameworks and the rising “right-to-suicide” debate in Taiwan

Ethical positions on suicide can be categorized as libertarian, relativist, and moralist [10, 11]. These perspectives affect people’s attitudes towards suicide prevention. From a libertarian standpoint, suicide is considered an individual right, and the government generally has no authority to intervene; in some of its versions, government should even actively legislate to provide assisted suicide services that respect the autonomy of persons who choose to end their own lives [11, 12]. Those who embrace the relativist position determine the permissibility based on contextual factors, such as culture and the nature of the situation. From a moralist perspective, suicide is a prohibited behavior, and at least 20 countries still criminally penalize suicide [13–15]. In the United States, some studies criticize the inconsistency between supporting physician aid-in-dying in terminal illnesses and advocating suicide prevention in psychiatry [16, 17]. These studies apply the concept of parity, arguing that if people have the right to request physician aid-in-dying for terminal illnesses, then those suffering from severe mental distress who retain decision-making capacity should also have this right [16, 17].

In Taiwan, similar ethical discussions have become critical as the libertarian viewpoints of the “right to suicide” become more prevalent. Since 2014, Taiwan has incorporated the Convention on the Rights of Persons with Disabilities (CRPD) into its domestic legal framework [18]. Article 12, Paragraph 4 of the CRPD mandates that safeguards to prevent abuse must ensure that “measures relating to the exercise of legal capacity respect the rights, will and preferences of the person. “ [19]. In line with the authoritative interpretation of the Committee on the Rights of Persons with Disabilities (the CRPD Committee), any coercive measures that violate the rights, will and preferences of suicidal persons are strictly prohibited [20]. A survey by the Taiwan Suicide Prevention Center showed that agreement with the statement “suicide is a personal right, and the decision should be up to the individual” increased from 49.6% in 2013 to 63.7% in 2020 [21, 22]. However, in 2022, a bill titled the Dignified Good Death Act, which sought to legalize assisted suicide and euthanasia, was proposed but ultimately failed [23]. The growing public support for suicide as a personal right, contrasted with the failure to pass the Dignified Good Death Act, highlights an ethical contradiction in Taiwan’s public opinion and legal framework regarding suicide and assisted dying.

### A Taiwanese cultural turn: from Confucian patriarchy to constitutional rights

When it comes to the justification of suicide, whether a person has a right to die by suicide or is rational to make a choice of suicide is the often-cited discourses in literature [24]. Based on the liberty perspective of the right to suicide, the government should not interfere with a person’s suicidal behavior. Suicide prevention measures are not justifiable. Furthermore, based on the claim perspective, a person can request the government to assist suicidal behavior. Legislation for physician-assisted suicide will be mandated [25].

Taiwan implemented its constitution in 1947, blending Western liberal constitutionalism (emphasizing liberty and democracy) with Confucian influences (emphasizing patriarchy, meritocracy, and filial piety) [26]. In early laws, Confucianism was the more dominant ideology, as exemplified by the old Civil Code, which prioritized paternal authority in cases of parental disagreement and highlighting the importance of traditional gender roles over gender equality. It was not until the 1990s, through various Constitutional Court Interpretations, that the protection of specific human rights was gradually incorporated into Taiwanese law. Examples include safeguarding married women’s freedom to choose their place of residence, married daughters’ inheritance rights, the legalization of same-sex marriage [26] and the incorporation of several international human rights treaties into Taiwan’s domestic law [27]. As the human rights protection movement is a relatively recent phenomenon, the Confucian legacy (such as the belief in protecting one’s body as received from parents) may continue to influence Taiwanese perspectives on human rights and laws. It is still an open question whether human rights discourses often have substantive impact on the legal policy stance of the public in Taiwan or serve more as an empty rhetoric function [28].

In a jurisdiction that respects a person’s rational autonomous choice, the government will interfere with a person’s suicidal behavior only when the person is deemed irrational. Neither should the rational person’s suicidal behavior be prevented, nor should a physician’s assistance to it penalized. This legal stance is called weak paternalism. In contrast, in a jurisdiction that adopts hard paternalism, the government can prevent suicide and penalize physician-assisted suicide regardless of the rationality of the suicidal person [24, 29].

### Research gap and study objectives

Despite the critical nature of these ethical issues, there is a paucity of research examining public attitudes toward legal policies related to suicide prevention and physician-assisted suicide, and how these attitudes are influenced by underlying beliefs about suicide. Prior studies in Taiwan have shown that about 90% of the population supports government-led suicide prevention initiatives [30], while more than 80% express support for physician-assisted suicide [31], revealing a complex and potentially contradictory set of public views regarding personal autonomy and state intervention.

To further explore these dynamics, our research team conducted a nationally representative survey in 2020, funded by the National Science Council, shortly after the implementation of both the Suicide Prevention Act and the Patient Right to Autonomy Act. The survey included a section titled “Human Rights and Attitudes Toward Suicide Prevention,” which was designed to investigate how beliefs about suicide—particularly whether it is considered a rational act or a personal right—influence public support for suicide prevention laws. It is important to note that our survey did not directly assess respondents’ familiarity with the Suicide Prevention Act or the Patient Right to Autonomy Act, so interpretations of alignment between public attitudes and specific laws should be made with this limitation in mind. Specifically, we aimed to explore (1) Taiwanese views on laws related to suicide prevention, and (2) whether individuals who believe that “people have the right to suicide” and that “suicide is a rational behavior” are less likely to support preventive legislation and more likely to oppose the criminalization of assisted suicide. By examining these relationships, our study seeks to inform the development of ethically coherent and socially acceptable suicide prevention strategies.

## Methods

### Research sample and data collection

A national survey was conducted in Taiwan between June 1st and July 31st, 2020, recruiting individuals aged 20 and above (Research ethics committee approval number: TCHIRB-10803013). The study employed a dual-frame approach, incorporating both landlines and mobile phones. Verbal informed consent was obtained from all participants before the survey took place. A total of 1,087 participants provided complete responses for analysis. Detailed survey methodology can be found in the prior publications [30, 32].

### Study variables

#### Main predictors: Suicide-related beliefs

Two beliefs about suicide were inquired: (Question 1) Do you agree that people have the right to suicide? This was referred to as the “Right to suicide belief” hereafter. (Question 2) Do you agree that suicide is irrational? This was referred to as the ‘Suicide rationality belief” hereafter. Responses included “strongly agree, agree, somewhat agree, disagree, strongly disagree, do not know, and refuse to answer.” Those who answered “strongly agree, agree, or somewhat agree” for question 1 were classified as believing people have the right to suicide. Those who answered “disagree” and “strongly disagree” for question 2 were classified as believing suicide is rational. Those who answered “do not know” or “refuse to answer” were excluded from the analysis.

#### Outcome variables: suicide prevention and assisted suicide legislation

Participants’ attitudes toward laws related to suicide prevention and physician-assisted suicides were evaluated by the following questions: (1) Do you agree that “behavior on the Internet that instigates or provokes people to engage in suicidal behavior should be punished?” (2) Do you agree that “the government should impose penalties on media for providing detailed portrayals of suicide methods and reasons for suicide”? (3) Do you agree that the government should impose penalties on media when they report information about the sale of toxic substances (such as paraquat) or other lethal suicide tools (such as guns)”? (4) Do you agree that “physicians who assist in the suicide of terminally ill patients (referring to those with serious injuries or illnesses that doctors have diagnosed as incurable, with medical evidence showing that death is unavoidable in the near future) should be legally penalized”? Responses included “strongly agree, agree, somewhat agree, disagree, strongly disagree, do not know, and refuse to answer.” Those who answered “strongly agree, agree, or somewhat agree” were classified as agreeing with the specific question. We excluded those who answered “do not know” or “refuse to answer” from the analysis.

#### Sociodemographic factors

Several sociodemographic variables were assessed: age (20–39 years, 40–59 years, and 60 years or above), sex, marital status (married, single, and other), educational level (junior high school and below, senior high school, college/university degree or higher), and employment status (employed, unemployed, students, homemakers, and retired).

#### Mental health status

Past history of suicidal thoughts and psychiatric services utilization were inquired about. The specific questions were: “Have you ever had suicidal thoughts? (Responses: Yes/No)” and “Have you ever used psychiatric services? (Responses: Yes/No)”.

### Analytic strategies

To ensure the representativeness of the data, the responses were weighted based on the distributions of sex, age, and place of residence in Taiwan. All analyses utilized the weighted data. We first presented the sociodemographic and mental health characteristics of the survey participants. Chi-square tests were conducted to compare the participants’ attitudes toward suicide prevention and physician-assisted suicide legislation based on these characteristics. Logistic regression analyses were employed to examine the associations between two types of suicide beliefs (right to suicide belief and suicide rationality belief) and their respective associations with attitudes toward laws related to suicide prevention and physician-assisted suicide (outcome variables). Additionally, the interaction between “right to suicide belief” and “suicide rationality belief” was assessed to test whether individuals who endorsed both beliefs were more strongly opposed to suicide prevention laws and laws that penalize physician-assisted suicide than those who endorsed only one or neither.

## Results

A total of 1,087 participants completed the survey. Table 1 presents the sociodemographic and mental health characteristics of the participants, along with the prevalence of support for suicide prevention and assisted suicide legislation.

**Table 1:** Suicide beliefs and participant characteristics in relation to suicide prevention and assisted-suicide legislation (Chi-square test)

	Total (*N* = 1087)			Behavior that instigates suicidal behavior should be punished#				Media that provide detailed portrayals of the suicide methods and reasons for suicide should be penalized #				Media that report information about the sale of toxic substances or other lethal suicide tools should be penalized #				Physicians who assist in the suicide of terminally ill patients should be legally penalized #			
				Weighted% (95%CI) = 93.7 (92.2, 95.2)				Weighted% (95%CI) = 84.9 (82.6, 87.1)				Weighted% (95%CI) = 85.3 (83.1, 87.4)				Weighted% (95%CI) = 29.4 (26.5, 32.4)			
	Weighted%	(95% CI)		Weighted%	(95% CI)		*p*	Weighted%	(95% CI)		*p*	Weighted%	(95% CI)		*p*	Weighted%	(95% CI)		*p*
**People have the right to suicide**							0.989				0.392				0.332				< 0.001
Yes	55.3	52.1	58.5	93.59	91.40	95.77		83.95	80.35	87.55		85.45	82.37	88.53		35.93	31.70	40.17	
No	44.7	41.5	47.9	93.61	91.29	95.93		85.99	82.95	89.02		87.64	84.49	90.80		22.98	18.81	27.15	
**Suicide is rational**							< 0.001				0.027				< 0.001				< 0.001
Yes	26.3	23.5	29.1	87.49	83.30	91.67		81.08	76.10	86.05		78.46	73.37	83.55		18.76	13.80	23.72	
No	73.7	70.9	76.5	95.67	94.18	97.15		86.91	84.41	89.40		88.36	86.02	90.71		33.08	29.51	36.65	
**Gender**							0.525				0.698				0.262				0.047
Male	49.6	46.6	52.7	93.21	91.04	95.39		84.43	81.30	87.57		83.99	80.87	87.10		32.44	28.20	36.67	
Female	50.4	47.3	53.4	94.19	92.11	96.27		85.32	82.12	88.52		86.50	83.43	89.57		26.54	22.55	30.53	
**Age**							0.064				0.670				0.449				0.070
Over 60 years	27.6	24.6	30.5	94.82	91.83	97.81		83.72	78.71	88.74		87.60	83.12	92.07		26.03	19.75	32.31	
40–59 years	38.2	35.4	41.1	95.18	93.29	97.07		86.15	83.08	89.22		84.80	81.62	87.98		27.43	23.37	31.49	
20–39 years	34.2	31.3	37.1	91.21	88.20	94.21		84.36	80.48	88.25		83.97	80.06	87.88		34.13	28.95	39.30	
**Marital status**							0.012				0.657				0.129				0.077
Married	68.4	65.5	71.3	95.25	93.66	96.83		85.23	82.57	87.88		86.63	84.11	89.14		28.17	24.76	31.58	
Single	25.2	22.5	27.9	90.03	86.33	93.73		84.92	80.36	89.48		81.40	76.56	86.24		29.91	23.88	35.95	
Others^1^	6.4	4.9	7.9	91.84	84.82	98.87		80.73	70.65	90.80		86.06	77.32	94.80		43.06	29.81	56.31	
**Educational attainment**							0.517				0.420				0.002				0.320
Junior high^2^	10.4	8.5	12.4	94.63	89.93	99.34		82.50	74.70	90.29		91.57	85.78	97.35		22.98	13.92	32.05	
Senior high	28.0	25.3	30.8	94.92	92.43	97.41		83.12	78.69	87.55		90.11	86.64	93.58		31.40	25.91	36.89	
College^3^	61.6	58.6	64.5	92.99	90.98	95.01		86.10	83.38	88.82		81.99	78.99	84.99		29.59	25.88	33.30	
**Occupation**							0.904				0.540				0.534				0.027
Employed	66.0	63.0	69.0	93.37	91.53	95.22		86.07	83.55	88.59		84.15	81.48	86.83		31.12	27.61	34.63	
Unemployed	2.3	1.4	3.2	96.75	90.44	100.00		90.61	77.87	100.00		94.17	83.12	100.00		46.55	24.64	68.46	
Students	3.0	1.8	4.1	95.80	87.74	100.00		79.94	63.99	95.88		87.44	74.16	100.00		40.54	21.03	60.06	
Homemakers	12.7	10.6	14.7	94.91	91.11	98.70		81.90	75.01	88.79		84.73	78.37	91.09		25.34	17.42	33.26	
Retired	16.0	13.6	18.5	93.26	88.92	97.59		82.31	75.46	89.15		88.78	83.33	94.24		20.13	12.76	27.50	
**Mental health status**																			
**Past suicide ideation**							0.658				0.369				0.533				0.049
Yes	22.6	20.1	25.2	93.08	89.80	96.37		86.76	82.34	91.18		86.57	82.03	91.11		24.04	18.27	29.81	
No	77.4	74.8	79.9	93.89	92.20	95.58		84.33	81.74	86.92		84.88	82.38	87.37		31.04	27.68	34.40	
**Past psychiatric services utilization**							0.087				0.341				0.393				0.035
Yes	12.7	10.7	14.8	97.12	94.24	100.00		87.74	81.95	93.54		87.85	81.94	93.77		21.24	13.90	28.58	
No	87.3	85.2	89.3	93.22	91.55	94.89		84.46	82.04	86.89		84.88	82.53	87.23		30.63	27.47	33.78	

Note: The first three outcome variables assessed support for media regulation and online-content control specifically targeting suicide-related information, thereby reflecting endorsement of suicide-prevention legislation. The fourth outcome variable assessed support for imposing legal penalties on physicians who assisted terminally ill patients in suicide; agreement with such penalties indicated opposition to physician-assisted-suicide legislation

The proportion was weighted with 95% confidence interval. Chi-squared tests were used to estimate differences in proportions

*p <.05, **P <.01, ***p <.001

1 Including participants who were divorced, separated, widowed, cohabited, or other statuses

2 Including participants with a junior high degree or lower

3 Including participants with a college degree or higher

# Individuals who answered strongly agree, agree, or somewhat agree

Overall, respondents strongly supported suicide prevention measures, with 93.7% (95% CI = [92.2, 95.2]) agreeing that “instigating suicidal behavior online should be punished,” slightly higher than the 84.9% (95% CI = [82.6, 87.1]) who agreed that “detailed media portrayals of suicide should be penalized” and the 85.3% (95% CI = [83.1, 87.4]) who agreed that “media reporting information about the sale of toxic substances or lethal suicide tools should be penalized.” In contrast, only 29.4% (95% CI = [26.5, 32.4]) agreed that “physicians assisting terminally ill patients’ suicide should be penalized.”

Regarding suicide beliefs, 55.3% (95% CI = [52.1, 58.5]) believed that suicide is a human right, while 26.3% (95% CI = [23.5, 29.1]) viewed suicide as rational. Additional logistic regression (Supplementary Table 1) was conducted to evaluate the relationship between sociodemographic variables and suicide beliefs, showing that the youngest age group (20–39 years), individuals in the “Others” marital status category, and those with higher educational attainment were more likely to hold the belief that “people have the right to suicide.” Single individuals and those with previous suicidal ideation were more inclined to agree that “suicide is rational,” whereas the unemployed were less likely to support this belief.

Individuals who agreed with the statement “people have the right to suicide” did not differ in their support for the three suicide prevention measures compared to those who did not share this belief (Table 1). However, respondents with the right-to-suicide belief showed a markedly higher prevalence of support for penalizing physician-assisted suicide in terminally ill patients (35.93%), compared to those who did not hold this belief (22.98%). Among individuals who endorsed the belief that “suicide is rational,” a statistically significantly lower proportion expressed support for the three suicide prevention measures and legal penalties for physician-assisted suicide.

Regarding other sociodemographic factors, the belief that “behavior that instigates suicidal behavior should be punished” was most prevalent among married individuals (95.25%). The belief that “media reporting information about the sale of toxic substances or lethal suicide tools should be penalized” was least prevalent among those with a college degree or higher (81.99%). The statement “physicians who assist in the suicide of terminally ill patients should be penalized” was most prevalent among males (32.44%), unemployed individuals (46.55%), those who have never thought of suicide (31.04%), and those who have never used psychiatric services (30.63%).

Table 2 presents the results of logistic regression analyses examining the associations between sociodemographic characteristics, suicide beliefs, and support for various suicide prevention and assisted suicide legislation. Those who endorsed the belief that people have the right to suicide did not significantly differ in their attitudes toward laws related to suicide prevention. However, supporters of the right to suicide were more inclined to believe that “physician-assisted suicide should be penalized” (aOR = 1.8, 95% CI = [1.3, 2.5], *p* <.001). Individuals who believed that suicide is rational tended to disagree with all three suicide prevention measures (adjusted odds ratios ranging from 0.35 to 0.63, *p* <.05 for all outcomes). They also disagreed with the statement that “physician-assisted suicide should be penalized” (aOR = 0.47, 95% CI = [0.32, 0.69], *p* <.001).

**Table 2:** Associations of suicide beliefs and participant characteristics with attitudes toward suicide prevention and assisted-suicide legislation

	Behavior that instigates suicidal behavior should be punished#				Media that provide detailed portrayals of the suicide methods and reasons for suicide should be penalized #				Media that report information about the sale of toxic substances or other lethal suicide tools should be penalized #				Physicians who assist in the suicide of terminally ill patients should be legally penalized #			
	Weighted% (95%CI) = 93.7 (92.2, 95.2)				Weighted% (95%CI) = 84.9 (82.6, 87.1)				Weighted% (95%CI) = 85.3 (83.1, 87.4)				Weighted% (95%CI) = 29.4 (26.5, 32.4)			
	aOR	(95% CI)		*p*	aOR	(95% CI)		*p*	aOR	(95% CI)		*p*	aOR	(95% CI)		*p*
**People have the right to suicide**																
Yes	1.28	0.66	2.47	0.461	1.18	0.79	1.76	0.434	0.97	0.63	1.50	0.900	1.80	1.30	2.50	< 0.001
No	1.0				1.0				1.0				1.0			
**Suicide is rational**																
Yes	0.35	0.21	0.60	< 0.001	0.63	0.43	0.94	0.022	0.53	0.36	0.78	0.001	0.47	0.32	0.69	< 0.001
No	1.0				1.0				1.0				1.0			
**Gender**																
Male	1.0				1.0				1.0				1.0			
Female	1.15	0.66	2.01	0.623	1.15	0.78	1.69	0.489	1.24	0.84	1.83	0.275	0.80	0.59	1.08	0.147
**Age**																
Over 60 years	1.0				1.0				1.0				1.0			
40–59 years	0.87	0.35	2.13	0.752	0.96	0.58	1.57	0.863	1.07	0.59	1.93	0.826	0.81	0.50	1.31	0.387
20–39 years	0.56	0.21	1.53	0.261	0.79	0.44	1.40	0.413	1.27	0.65	2.46	0.487	1.14	0.67	1.94	0.637
**Marital status**																
Married	1.0				1.0				1.0				1.0			
Single	0.48	0.24	0.94	0.033	0.94	0.58	1.51	0.785	0.64	0.40	1.01	0.055	0.80	0.55	1.18	0.262
Others^1^	0.56	0.20	1.54	0.262	0.74	0.36	1.49	0.396	0.77	0.35	1.70	0.523	2.14	1.18	3.88	0.012
**Educational attainment**																
Junior high^2^	1.0				1.0				1.0				1.0			
Senior high	1.03	0.36	2.96	0.951	0.98	0.51	1.89	0.951	0.79	0.34	1.81	0.571	1.55	0.84	2.86	0.163
College^3^	0.93	0.36	2.40	0.883	1.26	0.67	2.38	0.471	0.39	0.18	0.83	0.016	1.34	0.73	2.45	0.351
**Occupation**																
Employed	1.0				1.0				1.0				1.0			
Unemployed	2.23	0.30	16.82	0.436	1.55	0.34	7.11	0.570	2.91	0.39	21.61	0.298	2.08	0.81	5.39	0.130
Students	3.02	0.38	23.85	0.296	0.69	0.24	2.02	0.500	1.69	0.47	6.07	0.420	1.66	0.71	3.86	0.244
Homemakers	0.73	0.32	1.71	0.472	0.67	0.39	1.16	0.154	0.62	0.35	1.10	0.101	0.88	0.53	1.47	0.633
Retired	0.55	0.21	1.48	0.237	0.68	0.38	1.23	0.201	1.37	0.67	2.83	0.390	0.51	0.27	0.94	0.030
**Mental health status**																
**Past suicide ideation**	0.84	0.45	1.56	0.576	1.16	0.75	1.80	0.510	1.15	0.72	1.84	0.549	0.73	0.50	1.07	0.111
(ref: none)																
**Past psychiatric services utilization**	2.49	0.86	7.22	0.092	1.29	0.71	2.34	0.400	1.09	0.59	2.00	0.792	0.68	0.42	1.11	0.119
(ref: none)																

Note: The proportion was weighted with 95% confidence interval

*p <.05, **P <.01, ***p <.001

1 Including participants who were divorced, separated, widowed, cohabited, or other statuses

2 Including participants with a junior high degree or lower

3 Including participants with a college degree or higher

aOR: adjusting for sex, age, marital status, educational attainment, occupational category, mental health status

# Individuals who answered strongly agree, agree, or somewhat agree

Demographic factors play a less consistent role. Single individuals are less likely to support punishing behavior that instigates suicide, while those in the “Others” marital status category (divorced, widowed, etc.) are more supportive of legal penalties for physicians who assist in terminally ill patients’ suicides. Those with a college degree or higher educational attainment were less likely to support penalizing media reporting about lethal suicide tools. Retired individuals show less support for penalizing physician-assisted suicide in terminally ill patients. Although individuals with a history of suicidal ideation or prior use of psychiatric services were less likely to support penalties for physician-assisted suicide (Table 1), this association lost statistical significance after adjusting for sociodemographic factors (Table 2).

Table 3 evaluated the interaction between beliefs that people have the right to suicide and that suicide is rational. This interaction showed no statistically significant effects across the four outcome measures (*p* >.05 for all interactions), indicating that individuals who endorsed both beliefs did not differ in their support for three suicide prevention measures or penalizing physician-assisted suicide compared to those who endorsed only one of these beliefs.

**Table 3:** Interaction of two suicide beliefs and their association with suicide prevention and assisted-suicide legislation

	Behavior that instigates suicidal behavior should be punished#				Media that provide detailed portrayals of the suicide methods and reasons for suicide should be penalized #				Media that report information about the sale of toxic substances or other lethal suicide tools should be penalized #				Physicians who assist in the suicide of terminally ill patients should be legally penalized #			
	Weighted% (95%CI) = 93.7 (92.2, 95.2)				Weighted% (95%CI) = 84.9 (82.6, 87.1)				Weighted% (95%CI) = 85.3 (83.1, 87.4)				Weighted% (95%CI) = 29.4 (26.5, 32.4)			
	aOR	(95% CI)		*p*	aOR	(95% CI)		*p*	aOR	(95% CI)		*p*	aOR	(95% CI)		*p*
**People have the right to suicide**																
Yes	2.19	0.86	5.55	0.098	1.55	0.93	2.58	0.095	1.20	0.68	2.10	0.530	2.26	1.57	3.26	< 0.0001
No	1.0				1.0				1.0				1.0			
**Suicide is rational**																
Yes	0.54	0.23	1.27	0.159	0.95	0.49	1.87	0.886	0.57	0.29	1.14	0.113	0.71	0.39	1.30	0.268
No	1.0				1.0				1.0				1.0			
Interaction: People have the right to suicide * Suicide is rational	0.43	0.13	1.43	0.170	0.47	0.20	1.10	0.082	0.82	0.34	1.95	0.651	0.48	0.22	1.05	0.067

Note: The proportion was weighted with 95% confidence interval

*p <.05, **P <.01, ***p <.001

1 Including participants who were divorced, separated, widowed, cohabited, or other statuses

aOR: adjusting for sex, age, marital status, educational attainment, occupational category, mental health status

## Discussion

### Main findings

Our research revealed an overall high support for suicide prevention measures — 93.7% for punishing online instigation of suicide, 84.9% for penalizing detailed media reports of suicide, and 85.3% for penalizing media reporting about the sale of toxic substances or other lethal suicide tools — suggesting strong public endorsement of preventive policies. However, the markedly lower support (29.4%) for penalizing physicians who assist in the suicide of terminally ill patients highlights a distinction in public attitudes between general suicide prevention and specific physician-assisted suicide. For many people in Taiwan, suicide prevention and physician-assisted suicide may coexist under a feasible regulatory scheme. Similarly, countries permitting physician-assisted suicide, such as the Netherlands, Canada, and Australia, also implement their suicide prevention policies [33–35].

Our research hypothesized that individuals with the “right to suicide belief” and “suicide rationality belief” would be more likely to oppose both suicide prevention laws and legal penalties for physician-assisted suicide. However, although 55.3% of respondents supported a right to suicide, they did not show a significant difference in their attitudes towards suicide prevention laws and were paradoxically more supportive of penalizing physician-assisted suicide. A minority of the respondents (26.3%), namely those with the “suicide rationality belief,” tended to oppose suicide prevention laws and disagree with penalizing physician-assisted suicide. This discrepancy suggests that the “right to suicide belief” does not align straightforwardly with relevant policy support among Taiwanese people. Instead, the “suicide rationality belief” more directly shapes attitudes towards suicide-related policies in Taiwan. The lack of significant interaction effects between beliefs in the right to suicide and the rationality of suicide (Table 3) suggested that these beliefs, while related, operate independently in influencing attitudes towards suicide prevention and assisted suicide legislation. To summarize, in a strict sense, Taiwanese society is neither libertarian nor moralist, instead tending to adopt a relativist stance on issues related to suicide prevention and physician-assisted suicide.

### Sociodemographic factors and attitudes towards suicide prevention and assisted suicide legislation

Sociodemographic factors played a less consistent role in our research. Gender, age, and mental health status (past suicide ideation and past psychiatric service utilization) showed no significant associations with support for the Suicide Prevention Act and Assisted Suicide Legislation. More inclined to endorse the belief in suicide rationality, single individuals were less likely to support punishing behavior that instigated suicide. Prone to endorse a right to suicide, those with a college degree or higher educational attainment were less likely to support penalizing media reporting about lethal suicide tools. However, tending to endorse a right to suicide, those in the “Others” marital status category (divorced, widowed, etc.) were more supportive of penalizing physicians assisting in terminally ill patients’ suicide. Disposed to reject the rationality of suicide, unemployed individuals did not show more support for penalizing physicians assisting terminally ill patients. With less support for the legal penalties for physician assisted suicide, retired people did not show inclined normative stance on suicide. It seemed that, independent of the beliefs about a right to or rationality of suicide, the above sociodemographic factors had their own specific contributions to attitudes towards suicide prevention and physician-assisted suicide. This emphasized the importance of considering multiple factors in understanding and addressing public opinions on suicide-related policies.

### The suicide-rationality belief and attitudes toward suicide prevention and assisted suicide legislation

Despite 26.3% of respondents holding the suicide-rationality belief, 85–95% of participants in our study supported three measures adopted from the Suicide Prevention Act in Taiwan. These findings indicated that Taiwanese society tends to adopt a more interventionist approach to suicide prevention—consistent with Beauchamp and Childress’s concept of hard paternalism, which holds that intervention is justified even when individuals possess rationality and decision-making capacity [36]. Notably, the three preventive measures examined in this study limit access to suicide-related information; they influence choice by reducing the cognitive availability of pro-suicide content rather than coercing suicidal persons directly. This pattern also reflects support for libertarian paternalism—an approach that guides behavior while avoiding direct coercion of those at risk [37]. These different paternalistic logics suggest broad public approval for governmental intervention, even when individual autonomy is partially curtailed. Yet attitudes may shift when the target is an identified patient (such as compulsory psychiatric admission for individual patients with mental illness) rather than the general public.

In our study, consistent with our hypothesis, respondents who agreed that “suicide is rational” tended to oppose suicide prevention laws and to reject penalties for physician-assisted suicide. This consistent pattern suggests that views on the rationality of suicide had a significant influence on attitudes towards suicide-related policies, although not to the level of shifting the majority stance. Rationality of suicide could be a key concept to focus on or avoid reinforcing when promoting suicide prevention policies in Taiwan. Cross-national evidence in Asia is still scarce, which limits firm comparisons. A South Korean community survey, for example, reported that support for preventing suicide climbed from 74.8% in 2020 to 86.3% in 2021, yet offered no explanation into the beliefs driving this change [38]. Conversely, an online study in Japan found that seeing suicide as incomprehensible or unpredictable did not affect respondents’ willingness to pay for prevention [39]. These mixed results suggested that in Asian countries, similar normative beliefs in suicide can yield different policy preferences. Further nuanced empirical exploration of the differences between Taiwan, Asia, and the rest of the world is warranted.

Turning to physician-assisted suicide for the terminally ill, only a minority in either belief group favored penalizing physicians—18.8% among respondents who regarded suicide as rational and 33.1% among those who did not. This disparity suggested that factors other than rationality shape end-of-life policy preferences. One possible factor is the CRPD’s position that legal capacity is not contingent solely on rational deliberation [40, 41]. Another possibility is that, when confronting terminal illness, respondents place greater weight on principles of beneficence and compassion, leading them to oppose sanctions on physicians who provide physician-assisted suicide [42]. Although Taiwan has reported no criminal physician-assisted suicide cases for decades [43] and mental capacity has remained a key criterion for accessing physician-assisted suicide in most countries and states that permit it globally [12, 44], additional research is needed to validate these explanations and clarify how notions of rationality influence public views of physician-assisted suicide policy.

### The right-to-suicide belief and attitudes toward suicide prevention and assisted suicide legislation

Despite 55.3% of respondents supporting the human right to suicide, our research revealed that those who consider suicide a human right showed no significant difference in support for suicide prevention laws but were more supportive of penalizing physician-assisted suicide. In contrast, the Japanese internet survey showed that the idea of a right to suicide was associated with less willingness to pay for suicide prevention [39]. The discrepancy suggests that the belief in “human right to suicide” does not straightforwardly align with policy support in Taiwan, perhaps because the notion of “human rights” lacks consistent meaning in everyday discourse.

When human rights are discussed as a universal abstract value (i.e., rights to which everyone is entitled simply by being human), they are often uncontroversial. However, when human rights are defined at a concrete content level (e.g., in legislation), divergences frequently emerge [45, 46]. In Taiwan the phrase “human right to suicide” often serves a rhetorical rather than a normative function. The historical trajectory of rights education helps explain this gap. The Taiwanese government first established the Human Rights Education Committee in 2001, proposing various initiatives and guidelines [47]. However, human rights education in primary and secondary schools continued to face numerous criticisms into the 2010s, including a lack of qualified teachers, insufficient allocated teaching hours, and perceptions that the guidelines merely paid lip service to the universality of human rights [48]. Because rights literacy developed slowly, the public may lack shared criteria for applying abstract principles to concrete issues such as suicide prevention or physician-assisted suicide. Cross-national evidence supports this interpretation. A 2015 tri-country survey on end-of-life decisions found that Japan—with extensive history of discussions on end-of-life issues—favoured withdrawal of life-sustaining treatment over active euthanasia, whereas Korea and China showed the opposite preference [49]. The Taiwanese case more closely resembles the latter pattern, hinting that limited civic deliberation can leave concept of “human rights” a rhetorical ideal.

A cross-sectional survey conducted in Taiwan in 2022 revealed that 86.2% of respondents supported physician-assisted suicide for individuals with terminal illnesses, while 72.6% supported it for those with severe cognitive impairments. Younger generations (aged 20–39) expressed greater support compared to older generations (aged 60 and above) [31]. As shown in our supplementary table, younger respondents were more likely to endorse the right-to-suicide belief, reflecting a cultural shift in Taiwan from collective Confucianism to a more individualistic discourse on rights. Monitoring whether improved rights education eventually harmonizes abstract beliefs with concrete policy choices remains an important agenda. Further qualitative research is needed to explore more deeply the context to which people refer when discussing human rights and suicide.

### The interplay between human rights and rationality discourses

Traditional legal reasoning posits that a person should be allowed to perform an action if they have the right to do so and their decision is deemed rational. Accordingly, the right to suicide may be restricted in cases where a person is judged to lack rationality. However, our findings did not indicate a moderating effect of perceived rationality on the recognition or exercise of the right to suicide. This suggests not only the limitations of traditional legal frameworks in capturing culturally embedded understandings of human rights and rationality in the context of suicide, but also highlights the pressing need to engage and empower the public in bridging these conceptual and policy domains [41].

### Methodological factors and the misalignment between ethical stances and policy judgments

Beyond these cultural explanations, measurement factors may also account for the counter-intuitive finding. Previous research has demonstrated that survey respondents may provide seemingly incoherent answers to related questions. For instance, individuals may express support for expanding welfare programs while simultaneously endorsing tax reductions [50]. Such responses suggest that participants are offering authentic answers, yet without engaging in comprehensive deliberation across the full range of implications or trade-offs underlying their judgments. The “right-to-suicide” question was broad and abstract, whereas the physician-assisted-suicide item was concrete and legalistic (“legal penalties” for physicians). Cognitive-interview studies show that respondents readily endorse abstract autonomy statements but often shift to a protective stance when professional or legal responsibility is invoked—even if their underlying moral judgment has not changed [51]. In addition, framing effects could have amplified this divergence: because the physician-assisted-suicide item followed three penalty-focused suicide-prevention items, some respondents may have adopted a consistent “penalize-to-protect” response set. These methodological considerations caution against interpreting the observed association as a purely value-based inconsistency; measurement artifacts may also be at play. Future studies should incorporate cognitive pre-testing, randomized item order, and scenario-based probes to validate attitudes toward suicide rights and physician-assisted suicide in Taiwan.

### Strengths and limitations

This study’s strengths lie in its use of a nationally representative sample to explore the relationship between suicide beliefs and attitudes toward suicide prevention and physician-assisted suicide laws. The timely data collection, conducted immediately after the legislation captured real-time reactions to the newly enacted policy. Based on the analysis results, we have highlighted the complexities involved in drawing a boundary between suicide prevention and physician-assisted suicide within the frameworks of human rights and rationality discourses. However, the study has some limitations. Its cross-sectional nature limits causal inferences, and selection bias may exist because of possible differences between respondents and non-respondents. The analysis omitted several potentially influential sociodemographic factors, such as religious beliefs, economic status, cultural values, and family history of suicide. Lastly, among those supporting the right to suicide, individual understandings of what constitute “human rights” remained unexplored, necessitating further qualitative research. Future longitudinal research could provide valuable insights into the evolution of these attitudes and beliefs, particularly in response to evolving policies and public health interventions.

## Conclusions

Our study indicated that the majority of Taiwanese people endorsed suicide prevention and physician-assisted suicide legal policies simultaneously. However, the public’s concept of “people have the right to suicide” may be ambiguous or rhetorical, as endorsing this right did not directly translate into specific policy attitudes. Conversely, believing that “suicide is rational” correlated with disapproval of suicide prevention policies. For suicide prevention, it might be beneficial to avoid reinforcing the notion that suicide is rational. Further qualitative research is needed to explore Taiwanese people’s nuanced views on “human rights” and “rationality” regarding suicide. In addition, emphasizing contextual factors that contribute to suicidality is crucial. It warrants interventions regardless of perceived individual rationality and human rights through approaches such as libertarian paternalism (e.g., nudging) and diminishing the impact of socio-cultural determinants of suicide at the population level [4, 52]. At that time, an appropriate fuzzy boundary between suicide prevention and physician-assisted suicide may emerge through the active engagement with and empowerment of the public, following a comprehensive consideration of bio-psycho-socio-cultural factors. We believe this observation holds relevance not only for Taiwan, but also for Asia and the broader global community.

## Electronic Supplementary Material

Below is the link to the electronic supplementary material.

Supplementary Material 1

## Data Availability

The datasets used and analysed during the current study are available from the corresponding author on reasonable request.
