# Supplementary material for: The impact of suicide beliefs on support for suicide prevention and physician-assisted suicide
Source: BMC Psychiatry. 2025 Jul 9;25:689. doi: 10.1186/s12888-025-07112-8 (PMC12239293; doi:10.1186/s12888-025-07112-8)
Supplement: Supplementary file 1 — Supplementary Material 1 [file 12888_2025_7112_MOESM1_ESM.docx]

Supplementary table 1. Logistic regression analysis of the association of socio-demographic and mental health characteristics with suicide beliefs

|  | People have the right to suicide^#^ | | | | Suicide is rational | | | |
| --- | --- | --- | --- | --- | --- | --- | --- | --- |
|  | Weighted% (95%CI) | | | | Weighted% (95%CI) | | | |
|  | =55.3 (52.1 , 58.5) | | | | =26.3 (23.5 , 29.1) | | | |
|  | aOR | (95% CI) | | P value | aOR | (95% CI) | | P value |
| **Gender** |  |  |  |  |  |  |  |  |
| Male | 1.00 |  |  |  | 1.00 |  |  |  |
| Female | 1.09 | 0.81 | 1.45 | 0.576 | 1.02 | 0.75 | 1.39 | 0.905 |
| **Age** |  |  |  |  |  |  |  |  |
| Over 60 | 1.00 |  |  |  | 1.00 |  |  |  |
| 40-59 | 1.47 | 0.93 | 2.31 | 0.096 | 0.95 | 0.59 | 1.53 | 0.819 |
| 20-39 | 2.83 | 1.69 | 4.75 | <.0001 | 1.04 | 0.60 | 1.78 | 0.901 |
| **Marital status** |  |  |  |  |  |  |  |  |
| Married | 1.00 |  |  |  | 1.00 |  |  |  |
| Single | 1.44 | 0.98 | 2.11 | 0.061 | 1.50 | 1.02 | 2.20 | 0.040 |
| Others^1^ | 1.85 | 1.03 | 3.30 | 0.039 | 1.29 | 0.67 | 2.46 | 0.450 |
| **Education** |  |  |  |  |  |  |  |  |
| Junior high^2^ | 1.00 |  |  |  | 1.00 |  |  |  |
| Senior high | 1.43 | 0.81 | 2.51 | 0.218 | 0.78 | 0.41 | 1.46 | 0.430 |
| College^3^ | 1.73 | 1.00 | 2.99 | 0.048 | 1.39 | 0.76 | 2.54 | 0.285 |
| **Occupation** |  |  |  |  |  |  |  |  |
| Employed | 1.00 |  |  |  | 1.00 |  |  |  |
| Unemployed | 0.67 | 0.21 | 2.17 | 0.505 | 0.11 | 0.01 | 0.84 | 0.034 |
| Students | 1.43 | 0.49 | 4.16 | 0.516 | 1.11 | 0.46 | 2.71 | 0.816 |
| Homemakers | 0.65 | 0.40 | 1.05 | 0.078 | 1.03 | 0.61 | 1.72 | 0.925 |
| Retired | 0.64 | 0.38 | 1.09 | 0.099 | 1.22 | 0.70 | 2.12 | 0.485 |
| **Mental health** |  |  |  |  |  |  |  |  |
| Suicidal ideation | 1.35 | 0.93 | 1.97 | 0.112 | 1.47 | 1.02 | 2.10 | 0.038 |
| (ref: none) |  |  |  |  |  |  |  |  |
| Psychiatric services utilization (ref: none) | 0.83 | 0.54 | 1.29 | 0.410 | 0.71 | 0.43 | 1.17 | 0.177 |

Note: The proportion was weighted with 95% confidence interval (CI)

aOR: adjusting for sex, age, marital status, educational attainment, occupational category, mental health

1 Including participants who were divorced, separated, widowed, cohabited, or other statuses

2 Including participants with a junior high degree or lower

3 Including participants with a college degree or higher

# Individuals who answered strongly agree, agree, or somewhat agree
